# Integrating Parasitological and Entomological Observations to Understand Malaria Transmission in Riverine Villages in the Peruvian Amazon

**DOI:** 10.1093/infdis/jiaa496

**Published:** 2021-04-27

**Authors:** Angel Rosas-Aguirre, Marta Moreno, Diamantina Moreno-Gutierrez, Alejandro Llanos-Cuentas, Marlon Saavedra, Juan Contreras-Mancilla, Jose Barboza, Freddy Alava, Kristhian Aguirre, Gabriel Carrasco, Catharine Prussing, Joseph Vinetz, Jan E Conn, Niko Speybroeck, Dionicia Gamboa

**Affiliations:** 1 Research Institute of Health and Society, Université catholique de Louvain, Brussels, Belgium; 2 Instituto de Medicina Tropical Alexander von Humboldt, Universidad Peruana Cayetano Heredia, Lima, Peru; 3 Department of Infection Biology, London School of Hygiene and Tropical Medicine, London, United Kingdom; 4 Facultad de Medicina Humana, Universidad Nacional de la Amazonía Peruana, Loreto, Peru; 5 Facultad de Salud Pública y Administración, Universidad Peruana Cayetano Heredia, Lima, Peru; 6 International Centers of Excellence for Malaria Research-Amazonia, Laboratorios de Investigación y Desarrollo, Facultad de Ciencias y Filosofía, Universidad Peruana Cayetano Heredia, Lima, Peru; 7 Division of Infectious Diseases, Department of Medicine, University of California San Diego, La Jolla, California, USA; 8 School of Public Health, Department of Biomedical Sciences, State University of New York, Albany, New York, USA; 9 Wadsworth Center, New York State Department of Health, Albany, New York, USA; 10 Section of Infectious Diseases, Department of Internal Medicine, Yale School of Medicine, New Haven, Connecticut, USA; 11 Departamento de Ciencias Celulares y Moleculares, Facultad de Ciencias y Filosofía, Universidad Peruana Cayetano Heredia, Lima, Perú

**Keywords:** malaria, transmission, heterogeneity, Amazon, incidence, prevalence, human biting rate, entomological inoculation rate, Peru

## Abstract

**Background:**

Remote rural riverine villages account for most of the reported malaria cases in the Peruvian Amazon. As transmission decreases due to intensive standard control efforts, malaria strategies in these villages will need to be more focused and adapted to local epidemiology.

**Methods:**

By integrating parasitological, entomological, and environmental observations between January 2016 and June 2017, we provided an in-depth characterization of malaria transmission dynamics in 4 riverine villages of the Mazan district, Loreto department.

**Results:**

Despite variation across villages, malaria prevalence by polymerase chain reaction in March 2016 was high (>25% in 3 villages), caused by Plasmodium *vivax* mainly and composed of mostly submicroscopic infections. Housing without complete walls was the main malaria risk factor, while households close to forest edges were more commonly identified as spatial clusters of malaria prevalence. Villages in the basin of the Mazan River had a higher density of adult *Anopheles darlingi* mosquitoes, and retained higher prevalence and incidence rates compared to villages in the basin of the Napo River despite test-and-treat interventions.

**Conclusions:**

High heterogeneity in malaria transmission was found across and within riverine villages, resulting from interactions between the microgeographic landscape driving diverse conditions for vector development, housing structure, and human behavior.

The history of malaria in Peru shows that important reductions in the malaria burden can be obtained with intensive and comprehensive standard control measures, but also that this progress can quickly be lost if there is not a long-term country plan to consolidate the achievements and prevent malaria resurgence [1]. For instance, the Global Fund-sponsored PAMAFRO project supported the strengthening of microscopic diagnosis, the implementation of active test-and-treat interventions, and the distribution of long-lasting insecticidal mosquito nets (LLINs), with a community-based intercultural approach, between 2005 and 2010 in the Peruvian Amazon. During this period, malaria declined drastically in the most affected department of Loreto from 54 291 reported cases (25% due to *Plasmodium falciparum*) in 2005 to 10 504 cases (20% due to *P. falciparum*) in 2010 [1, 2]. Regrettably, the marked reduction of international donors’ support and the unusually heavy rains that caused floods in riverine villages starting in 2012, led to a 5-fold increase in cases between 2010 and 2015 (60 302 cases) [1, 3].

The political and financial commitment from the Peruvian government was crucial not only to respond to this malaria resurgence, but also to establish a long-term initiative called Plan Malaria Cero started in 2017, with the ambitious aim of eliminating malaria in the country by 2036 [4]. Intensive malaria control interventions based on the PAMAFRO experience in high-risk malaria villages contributed to a substantial reduction in the malaria incidence in Loreto, reporting 22 037 cases in 2019 [5]. As transmission decreases, malaria strategies need to be more focalized and adapted to the local malaria epidemiology determined by the complex interactions among *Plasmodium* parasites, human behavior, and highly variable environments driving changes in mosquito vector behavior and habitat suitability [1, 6, 7].

Rural riverine villages, characterized by poverty and limited access to health facilities, concentrate an important proportion of reported malaria cases in Loreto [8]; however, transmission dynamics in these villages remain understudied [9, 10]. By accounting for the ecological heterogeneity of 2 distinctive river basins, we identified 4 riverine villages (2 on each river) in the endemic district of Mazan in Loreto department and calculated the prevalence of malaria infections by quantitative real-time PCR (qPCR) in March and September 2016, and March 2017. These parasitological measurements were then related to incidence rates of reported malaria episodes between January 2016 and June 2017, and to available entomological and environmental observations in the same period [11, 12], to better characterize malaria transmission in these villages.

## METHODS

### Study Area

The district of Mazan is home to approximately 13 900 inhabitants, with one-third of them living in Mazan town near the confluence of the Napo River and its tributary the Mazan River about 50 km from Iquitos city (capital of Loreto). Mazan was among the districts in Loreto with the highest annual parasite index in 2017 (annual parasite incidence, 96.4 cases per 1000 inhabitants; *P. vivax*, 1066 cases; *P. falciparum,* 374 cases) [5, 13]. Most cases (>80%) occurred in approximately 20 villages located in the Mazan and Napo River basins. The exophilic, exophagic, and anthropophagic *Anopheles darlingi* is the main malaria vector [12]. The primary interventions prior to this study were routine passive case detection, seasonally indoor residual spraying, and the delivery of LLINs in the first months of 2016.

### Design and Selected Villages

**Figure 1.**
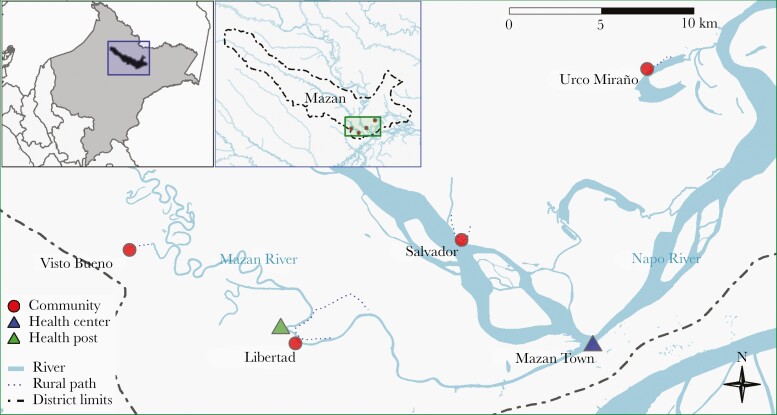
Study area, 4 riverine villages in Mazan district, Loreto department.

This study integrated available sociodemographic, parasitological, entomological and environmental data from Libertad (LIB) (3.496°S, 73.234°W) and Visto Bueno (VIB) (3.449°S, 73.317°W) in the Mazan basin, and Salvador (SAL) (3.445°S, 73.154°W) and Urco Miraño (URC) (3.361°S, 73.064°W) in the Napo basin (Figure 1 and Supplementary Table 1).

The Direction of Loreto (477–2016), Universidad Peruana Cayetano Heredia (UPCH, 64746), and WHO Ethics Review Committee (0002669) approved entomological collections, while UPCH (66235) approved sociodemographic and parasitological surveys.

### Baseline Data

Households were censused, georeferenced, and updated during surveys in March and September 2016 and March 2017. Mobile devices programmed with Open Data Kit allowed for the collection of sociodemographic and epidemiological data, including questions about LLIN use, and the time to go to sleep and wake up.

### Prevalence Data

Malaria prevalence by both microscopy and qPCR were estimated from datasets of a pilot study that assessed the effects and costs of population-wide malaria test-and-treat interventions (in addition to passive case detection) before the high malaria transmission season in the Peruvian Amazon [13, 14]. Visits to all censused households in VIB and URC were made in March and September 2016 and March 2017. Available household members in March 2016 and March 2017 had axillary temperature taken and history of fever or any other malaria-compatible symptom documented. Finger-prick blood samples were collected for immediate microscopy in the field and dried blood spots for later analysis by qPCR. Health workers from the Ministry of Health accompanied personnel conducting field research activities, and treated microscopically confirmed infections regardless of symptoms according to national guidelines [15]. The same procedures were carried out in September 2016, but microscopic diagnosis was not performed by the pilot study; individuals with any malaria-compatible symptom were referred to health facilities for diagnosis and treatment.

Additional household visits in LIB and SAL were made in April 2016 and April 2017 (30 days after visits in March). A supplementary intervention in these villages consisted of making qPCR results available within 3 days of the sample collection in March and April of 2016 and 2017, allowing for the treatment of qPCR-positive individuals who were negative in an initial microscopic test. These qPCR tests were processed at the local research laboratory in Iquitos (Supplementary Material).

### Incidence Data

Passive case-detection data of microscopically confirmed malaria episodes between January 2016 and June 2017 were obtained from the Ministry of Health surveillance systems [5] and verified with data registered at health facilities. Monthly incidence rates were calculated as the number of registered episodes (× 100) divided by the number of individuals censused during the baseline survey. In cases of recurrent registered episodes, intervals ≥ 60 days for *P. vivax* and ≥ 30 days for *P. falciparum* were used to identify independent episodes [16].

### Entomological and River Level Data

Published data of indoor and outdoor 12-hour mosquito collections by human landing catch were used to estimate human biting rates (HBRs), the hourly biting behavior of *An. darlingi*, and entomological inoculation rates (EIRs) in March, June, September, and November 2016, and March 2017 [12]. Available vector and human behavior data were used to provide a rough estimate of the proportion of human exposure to *An. darlingi* occurring indoors, in scenarios in which people are or are not protected by LLIN, as described by Monroe et al [17]. Larval sampling data from water bodies located by ground inspection within 1 km of each village (September and November 2016, and March 2017) identified *An. darlingi* breeding sites [11]. Daily water levels of the Napo River measured by a hydrological station near the confluence of the Napo and Mazan rivers were averaged monthly [18].

### Data Analysis

Baseline characteristics between villages were compared using the *χ*^2^ test. Trend charts related monthly incidence rates, malaria prevalence, HBRs, EIRs, and river levels. Uni- and multivariate mixed-effects logistic regression models determined risk factors for malaria infection by qPCR in March 2016. The following potential risk factors were assessed as fixed effects: age, sex, occupation, malaria antecedent, LLIN ownership, LLIN use, and housing structure. Random effects accounted for individuals nested within households, and households nested within villages. Factors with *P* values < .2 for the Wald test in the univariate analysis were included in the full multivariate model. Manual backward elimination guided by the minimization of the Akaike information criterion reduced of the number of factors in final models. Likelihood ratio tests compared nested models.

Households were mapped and their age-standardized malaria prevalence estimated considering the population’s age structure. Global Moran index (I) assessed the spatial autocorrelation of malaria prevalence in villages. Local Moran I with correction by false discovery rate assessed the variation in spatial autocorrelation over the village area, allowing for the classification of each household into 5 categories: high-high (a high prevalent household surrounded by high prevalent households), low-low (a low prevalent household surrounded by low prevalent households), outliers high-low and low-high, and not significant. A maximum neighborhood distance criterion of 550 meters was chosen for LIB, URC, and SAL [10], whereas it was set at 200 meters for VIB given the smaller distance between households. Malaria prevalence was weighted according to the inverse of the distance to the neighboring house. All analysis and maps were done using R version 2.15 software (R Development Core Team, R Foundation for Statistical Computing).

## RESULTS

A total of 205 households and 1015 residents were censused and distributed as follows: LIB (60 households, 297 individuals), VIB (15 households, 60 individuals), URC (53 households, 284 individuals), and SAL (77 households, 374 individuals) (Supplementary Table 1). The overall ratio of female to male was 0.9, with most individuals aged younger than 20 years (median, 16.2). Secondary or higher education level was higher in URC (28.7%) than in other villages (approximately 10%) (*P* < .001). Forest-related economic activities predominated (31.2%–41.7%) with no differences between villages. Compared to LIB and SAL, residents in URC and VIB more frequently reported malaria antecedent in the past year as well as appropriate LLIN household coverage (*P* < .01). LLIN use the previous night ranged between 75.3% and 81.4%, with no differences across villages. Housing structures without complete wooden walls were common (63.2%), especially in LIB (73.7%) (*P* < .001). In contrast to VB and LIB, in URC and SAL, tin frequently replaced palm leaves as ceiling material and more participants reported having electricity at home (*P* < .001) (Supplementary Table 2 and Supplementary Table 3). This latter finding was negatively associated with the proportion of individuals sleeping at 20:00 hours in URC (75%) and SAL (70%) in comparison with those in VB (88%) and LIB (96%) (*P* < .001).

**Figure 2.**
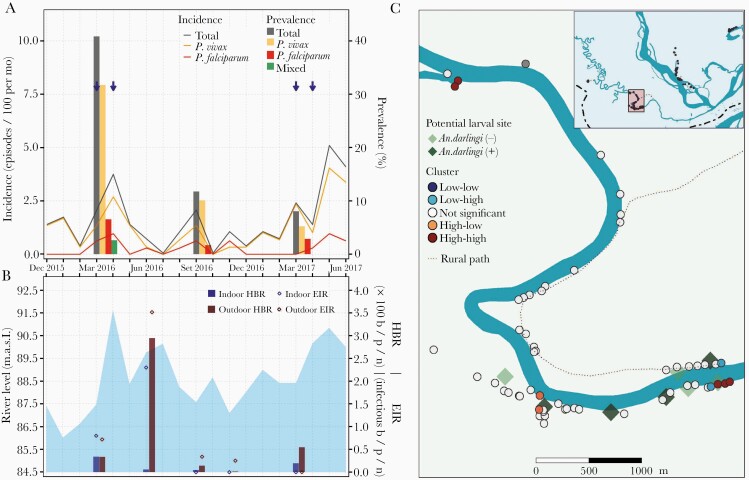
Parasitological, entomological, and river-level observations between January 2016 and June 2017, and spatial clusters of malaria prevalence in March 2016 in Libertad village. *A*, Evolution of malaria prevalence and incidence rates: Arrows indicate months when test-and-treat interventions were conducted with polymerase chain reaction (PCR) result available for malaria treatment within 3 days of the sample collection. *B*, Bars represent human biting rates (HBR) and points entomological inoculation rates (EIR) estimated in March, June, September and November 2016 and in March 2017. Shaded area represents monthly water levels of the Napo River. *C*, Spatial distribution of households classified according to local Moran I as high-high, high-low, not significant, low-high, and low-low clusters of malaria prevalence in March 2016. Abbreviations: b/p/n, bites/person/night; MASL, meters above sea level.

**Figure 3.**
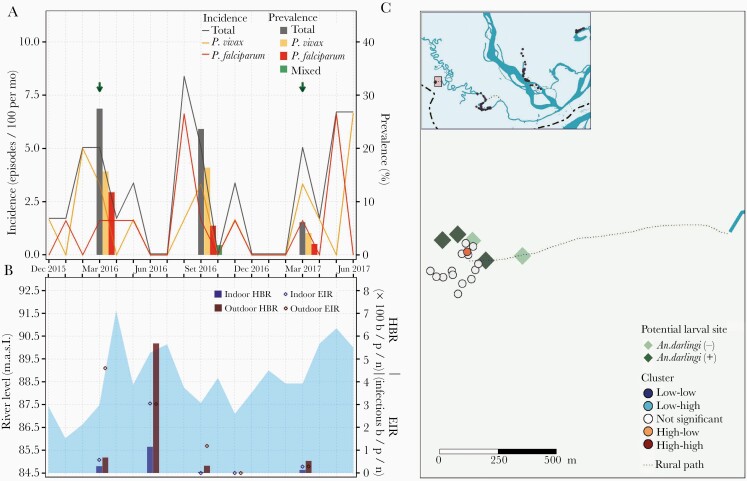
Parasitological, entomological, and river level observations between January 2016 and June 2017, and spatial clusters of malaria prevalence in March 2016 in Visto Bueno village. *A*, Evolution of malaria prevalence and incidence rates: Arrows indicate months when test-and-treat interventions were conducted using microscopy. *B*, Bars represent human biting rates (HBR) and points entomological inoculation rates (EIR) estimated in March, June, September, and November 2016 and in March 2017. Shaded area represents monthly water levels of the Napo River. *C*, Spatial distribution of households classified according to local Moran I as high-high, high-low, not significant, low-high, and low-low clusters of malaria prevalence in March 2016. Abbreviations: b/p/n, bites/person/night; MASL, meters above sea level.

**Figure 4.**
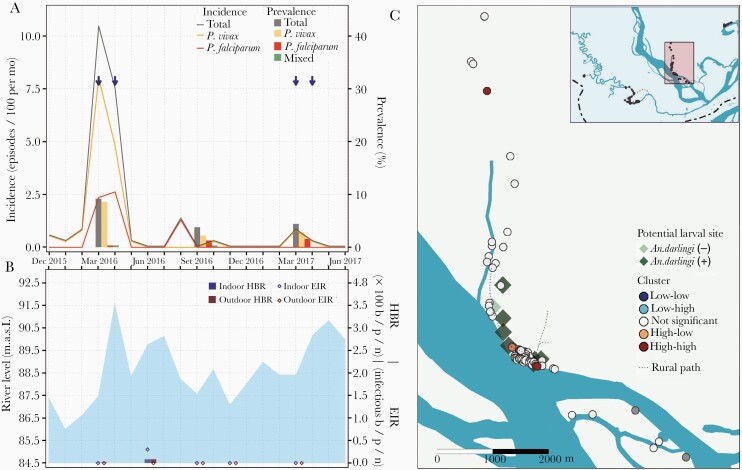
Parasitological, entomological, and river level observations between January 2016 and June 2017, and spatial clusters of malaria prevalence in March 2016 in Salvador village. *A*, Evolution of malaria prevalence and incidence rates: Arrows indicate months when test-and-treat interventions were conducted with polymerase chain reaction (PCR) result available for malaria treatment within 3 days of the sample collection. *B*, Bars represent human biting rates (HBR) and points entomological inoculation rates (EIR) estimated in March, June, September, and November 2016 and in March 2017. Shaded area represents monthly water levels of the Napo River. *C*, Spatial distribution of households classified according to local Moran I as high-high, high-low, not significant, low-high, and low-low clusters of malaria prevalence in March 2016. Abbreviations: b/p/n, bites/person/night; MASL, meters above sea level.

**Figure 5.**
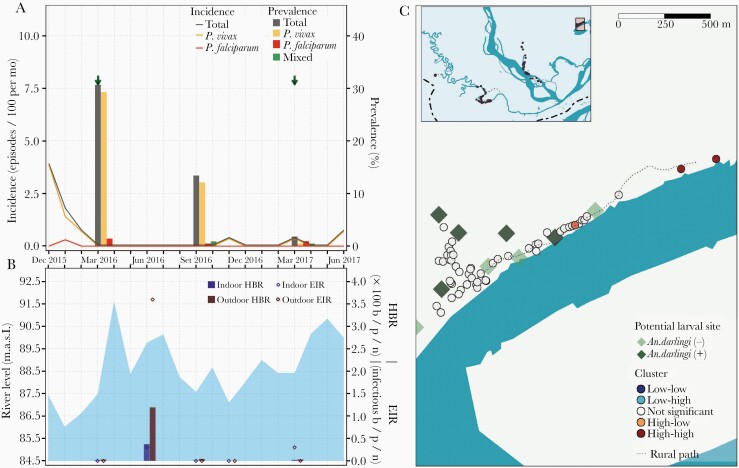
Parasitological, entomological, and river level observations between January 2016 and June 2017, and spatial clusters of malaria prevalence in March 2016 in Urco Miraño village. *A*, Evolution of malaria prevalence and incidence rates: Arrows indicate months when test-and-treat interventions were conducted using microscopy. *B*, Bars represent human biting rates (HBR) and points entomological inoculation rates (EIR) estimated in March, June, September, and November 2016 and in March 2017. Shaded area represents monthly water levels of the Napo River. *C*, Spatial distribution of households classified according to local Moran I as high-high, high-low, not significant, low-high, and low-low clusters of malaria prevalence in March 2016. Abbreviations: b/p/n, bites/person/night; MASL, meters above sea level.

Samples for qPCR analysis were available for 823 individuals in March 2016, 843 in September 2016, and 800 in March 2017. Malaria prevalence was the highest in March 2016 for all villages (LIB, 40.9% [95% confidence interval, CI, 34.5%–47.5%]; VIB, 27.5% [95% CI, 16.3%–42.0%]; SAL, 9.2% [95% CI, 6.4%–13.0%]; and URC, 30.7% [95% CI, 24.7%–37.4%]), with *P. vivax* to *P. falciparum* ratios (LIB, 5:1; VIB, 1.3:1; SAL, 28:1; and URC, 21:1) and submicroscopic to microscopic infection ratios (LIB, 93:1; VIB, 13:1; SAL, 29:1; and URC, 16:1) varying widely across villages (Table 1, Figure 2A, Figure 3A, Figure 4A, and Figure 5A). Submicroscopic mixed infections were found in LIB (6 infections) and SAL (1 infection). The prevalence in September 2016 decreased for all villages (LIB, 11.8% [95% CI, 8.1%–16.7%]; VIB, 23.6% [95% CI, 13.7%–37.3%]; SAL, 3.8% [95% CI, 2.0%–6.7%]; and URC, 13.4% [95% CI, 9.4%–18.7%]), but the *P. vivax* to *P. falciparum* ratio continued to fluctuate across villages (LIB, 6:1; VIB, 3:1; SAL, 2:1; and URC, 28:1). The lowest malaria prevalence, composed of mainly submicroscopic infections, were found in March 2017 (LIB, 8.1% [95% CI, 4.9%–12.8%]; VIB, 6.1% [95% CI, 1.6%–17.9%]; SAL, 4.4% [95% CI, 2.5%–7.4%]; and URC, 1.8% [95% CI, 0.6%–4.9%]) with *P. vivax* to *P. falciparum* infection ratios ranging between 1:2 and 2:1.

**Table 1.** Prevalence of Malaria by qPCR in March 2016, September 2016, and March 2017

	March 2016		September 2016a		March 2017	
Infection Detected	n (%)	95% CI	n (%)	95% CI	n (%)	95% CI
Libertad						
Total samples (n)	230		238		211	
Overall	94 (40.9)	34.5–47.5	28 (11.8)	8.1–16.7	17 (8.1)	4.9–12.8
*P. vivax*	73 (31.7)	25.9–38.2	24 (10.1)	6.7–14.8	11 (5.2)	2.8–9.4
Submicroscopic	72 (31.3)	25.5–37.8	…	…	7 (3.3)	1.5–7.0
Microscopic	1 (0.4)	.0–2.8	…	…	4 (1.9)	.6–5.1
*P. falciparum*	15 (6.5)	3.8–10.7	4 (1.7)	.5–4.5	6 (2.8)	1.2–6.4
Submicroscopic	15 (6.5)	3.8–10.7	…	…	6 (2.8)	1.2–6.4
Microscopic	0 (0.0)		…	…	0 (0.0)	
Mixed	6 (2.6)	1.1–5.9	0 (0.0)		0 (0.0)	
Visto Bueno						
Total samples (n)	51		55		49	
Overall	14 (27.5)	16.3–42.0	13 (23.6)	13.7–37.3	3 (6.1)	1.6–17.9
*P. vivax*	8 (15.7)	7.5–29.1	9 (16.4)	8.2–29.3	2 (4.1)	.7–15.1
Submicroscopic	8 (15.7)	7.5–29.1	…	…	2 (4.1)	.7–15.1
Microscopic	0 (0.0)				0 (0.0)	
*P. falciparum*	6 (11.8)	4.9–24.6	3 (5.5)	1.4–16.1	1 (2.0)	.1–12.2
Submicroscopic	5 (9.8)	3.7–22.2	…	…	1 (2.0)	.1–12.2
Microscopic	1 (2.0)	.1–11.8	…	…	0 (0.0)	
Mixed	0 (0.0)		1 (1.8)	.1–11.0	0 (0.0)	.2–9.1
Salvador						
Total samples (n)	327		319		318	
Overall	30 (9.2)	6.4–13.0	12 (3.8)	2.0–6.7	14 (4.4)	2.5–7.4
*P. vivax*	28 (8.6)	5.9–12.3	7 (2.2)	1.0–4.7	9 (2.8)	1.4–5.5
Submicroscopic	27 (8.3)	5.6–11.9	…	…	7 (2.2)	1.0–4.7
Microscopic	1 (0.3)	.0–2.0	…	…	2 (0.6)	.1–2.5
*P. falciparum*	1 (0.3)	.0–2.0	4 (1.3)	.4–3.4	5 (1.6)	.6–3.8
Submicroscopic	1 (0.3)	.0–2.0	…	…	5 (1.6)	.6–3.8
Microscopic	0 (0.0)		…	…	0 (0.0)	
Mixed	1 (0.3)	.0–2.0	1 (0.3)	.0–2.0	0 (0.0)	
Urco Miraño						
Total samples (n)	215		231		222	
Overall	66 (30.7)	24.7–37.4	31 (13.4)	9.4–18.7	4 (1.8)	.6–4.9
*P. vivax*	63 (29.3)	23.4–35.9	28 (12.1)	8.3–17.2	1 (0.5)	.0–2.9
Submicroscopic	59 (27.4)	21.7–34.0	…	…	1 (0.5)	.0–2.9
Microscopic	4 (1.9)	.6–5.0	…	…	0 (0.0)	
*P. falciparum*	3 (1.4)	.4–4.4	1 (0.4)	.0–2.8	2 (0.9)	.2–3.6
Submicroscopic	3 (1.4)	.4–4.4	…	…	2 (0.9)	.2–3.6
Microscopic	0 (0.0)		…	…	0 (0.0)	
Mixed	0 (0.0)		2 (0.9)	.2–3.4	1 (0.5)	.0–2.9

Abbreviations: CI, confidence interval; P. *falciparum, Plasmodium falciparum; P. vivax, Plasmodium vivax*; qPCR, quantitative polymerase chain reaction.

aMicroscopic diagnosis not performed.

In all villages, HBRs reached the highest levels 2 months after the peak of the Napo River (June 2016) (Figure 2B, Figure 3B, Figure 4B, and Figure 5B), and were followed by an increase in incidence in August–September 2016 (second peak) of predominantly *P. vivax* episodes in LIB and *P. falciparum* episodes in VIB and SAL. Only in VIB, the highest EIR was found in March 2016, not coinciding with the highest HBR. In comparison with villages in the Napo basin (SAL and URC), villages in the Mazan basin (LIB and VIB) had higher HBR and EIR levels, and maintained higher prevalence and incidence rates despite test-and-treat interventions. In contrast to the highest HBR levels outside houses in most surveys, differences between outdoor and indoor EIR values were less obvious. About 90% of human exposure to *An. darlingi* bites between 18:00 and 6:00 hours occurred indoors where LLINs were not available and/or used, according to the matched analysis of mosquito and human behavior data. The current LLIN usage in villages would prevent approximately 60% of human exposure (Supplementary Table 4).

The multivariate model for malaria infection by qPCR in March 2016 confirmed the influence of clustered data at household and village level, and the increased risk for individuals living in houses with incomplete walls (adjusted OR [aOR], 1.7; 95% CI, 1.1–2.6) (Table 2). Malaria prevalence was also slightly higher in individuals between 10 and 29 years old (aOR, 1.5; 95% CI, 1.0–2.3; *P* = .073) and men (aOR, 1.4; 95%CI, 1.0–2.0; *P* = .067) compared respectively with younger children and women, but not significantly.

**Table 2.** Uni- and Multivariate Risk Factor Analysis for Malaria Infection in March 2016

Risk Factor	n/N %	OR	OR 95% CI	aOR	aOR 95% CI
Age group, y					
<10	60**/** 290 (20.7)	Ref			
10–29.9	70**/** 243 (28.8)	1.5**	.9–2.2	1.5**	1.0–2.3
≥30	74**/** 290 (25.5)	1.2	.8–1.8	1.2	0.8–1.8
Sex					
Female	89**/** 399 (22.3)	Ref			
Male	115**/** 424 (27.1)	1.3	.9–1.9	1.4**	1.0–2.0
Occupation					
Not forest related	128**/** 515 (24.9)	Ref			
Forest related	76**/** 308 (24.7)	1.0	.7–1.4		
Malaria in the past year					
No	90**/** 395 (22.8)	Ref			
Yes	111**/** 413 (26.9)	1.3	.9–1.9		
Sleep under a LLIN the previous night					
Yes	154**/** 640 (24.1)	Ref			
No	48**/** 176 (27.3)	1.2	.8–1.9		
1 LLIN for 2 people					
Yes	89**/** 390 (22.8)	Ref			
No	115**/** 433 (26.6)	1.3	.9–1.9		
Indoor residual spray in past year					
Yes	154**/** 484 (31.8)	Ref			
No	49**/** 336 (14.6)	0.9	.5–1.5		
Number of external walls in house					
Complete, 4	52**/** 288 (18.1)	Ref			
None/incomplete, 0–3	152**/** 535 (28.4)	1.6*	1.1–2.5	1.7*	1.1–2.6
Ceiling material					
Tin, calamina	63/ 283 (22.3)	Ref			
Palm leaves	141/ 540 (26.1)	1.0	.6–1.6		
Electricity available					
Yes	74/ 345 (21.4)	Ref			
No	130/ 478 (27.2)	0.8	.5–1.3		
Distance to *Anopheles darlingi* larval site					
≥ 200 meters	140/ 485 (28.9)	Ref			
< 200 meters	63/ 314 (20.1)	0.8	.5–1.2		

Abbreviations: aOR, adjusted odds ratio; CI, confidence interval; n, number of malaria infected people; N, total number of people; OR, odds ratio; Ref, reference.

* *P* < .05, **P < .01

Global Moran I confirmed spatial autocorrelation of age-standardized household prevalence in March 2016 in 2 villages: LIB (I = 0.13, *P* = .04) and URC (I = 0.09, *P* = .04). Local Moran I identified high-high households (high-risk clusters) in LIB, URC, and SAL, but not in VIB (Figure 2C, Figure 3C, Figure 4C, and Figure 5C). High-risk clusters in LIB and URC were, respectively, 5 and 2 of the outermost households in the villages, while in SAL this included 2 households in the most populated area and 1 outermost household near a narrow river gorge that joins the Napo River. In addition, high-low outliers (high prevalent households surrounded by low prevalent households) were identified in all villages, located mainly in the most populated areas. High-risk clusters were not closer to *An. darlingi* larval sites when compared with other households (*P* > .05).

## DISCUSSION

The combined analysis of parasitological, entomological, and environmental observations between January 2016 and June 2017 allowed for an in-depth characterization of malaria transmission dynamics in riverine villages in Mazan, an Amazonian district where poverty, limited accessibility to health care, and village-specific ecology create challenges for malaria control. Despite some degree of heterogeneity across villages, malaria prevalence in the survey in March 2016 was high (> 25% in 3 villages), comprising mostly *P. vivax* submicroscopic infections. Whereas the housing without complete walls was the main risk factor for malaria, the proximity to *An. darlingi* breeding sites was not associated with malaria. Interestingly, villages in the Mazan basin had the highest entomological indicators and maintained the highest prevalence and incidence rates among study villages despite test-and-treat interventions.

The earlier high malaria incidence rates in LIB, VIB, and SAL in March–April 2016 in comparison to historically seasonal patterns of malaria incidence in the Mazan district (with peaks usually in June–July) are mainly explained by increased test-and-treat efforts in those months as part of a concurrent intervention pilot study. These efforts may have affected the temporal dynamics of malaria transmission, bringing a second peak of malaria incidence in these villages 2–3 months after their highest HBR levels in June 2016. The submicroscopic parasite carriers may have contributed to a silent infectious reservoir able to maintain mild-moderate transmission, despite population-wide malaria testing and treatment of confirmed infections in March–April of 2016 and 2017, and the drop in vector-human contact rates in the dry season of 2016. Indeed, several cross-sectional surveys in the Peruvian Amazon have consistently observed that a majority of *P. vivax* and *P. falciparum* infections could only be detected by sensitive molecular tests, suggesting the important role of these submicroscopic infections in sustaining malaria transmission [19–21]. For instance, a recent cohort study that screened full populations for malaria in riverine endemic villages showed that most low-density infections escaped standard surveillance using microscopy, even if residents were consecutively screened at intervals of 10 days over a month [14].

The persistently higher prevalence and incidence rates in the Mazan basin compared to those in the Napo basin (regardless of the type of test-and-treat intervention implemented) were in agreement with higher HBRs and EIRs in Mazan villages compared with the Napo ones. Contrary to what was expected, *An. darlingi* breeding sites in the Mazan basin were not the most numerous and densely inhabited by larvae among the study villages. Indeed, our group recently showed that SAL (Napo basin) was the village with the greatest number of *An. darlingi* larvae and anopheline larvae in general during the same study period, despite having the lowest density of adult mosquitoes [11, 12]. Notably, the presence and density of mosquito larvae in aquatic habitats and consequently the number of adults capable of malaria transmission are regulated by a variety of ecosystem processes operating and interacting at several organizational levels and spatial and temporal scales [22]. Understanding the patterns in the productivity of larval habitats requires knowledge about the local interactions between abiotic (eg, hydrology, temperature, shade, pH, salinity, and nutrients) and biotic (eg, predation and competition) factors [23–25]. A recent study of larval habitats in Mazan and Napo river watersheds found a correlation between *An. darlingi* larval sites and low forest coverage, low sunlight, and emergent vegetation [11]. Biotic factors, however, such as larval competition remain understudied [26]; it would be interesting to know, for instance, whether the proportion of *An. darlingi* larvae among total anopheline larvae (higher in water bodies in LIB and VIB than that in SAL) is a better determinant of the abundance of adult mosquitoes than larval densities (higher in SAL than in LIB and VIB).

The microgeographic landscape composition may also be an important determinant of the productivity of larval breeding sites, the abundance of adult mosquitoes, and ultimately malaria transmission in villages [26]. A recent study by our group, with high-resolution imagery captured by drones, showed remarkably heterogeneous land cover composition in study villages, suggesting a high diversity of locations at a microgeographical scale where *An. darlingi* can breed [27]. Published orthomosaics from this imagery [27] together with additional field visits confirmed that all outermost households classified as spatial clusters of high malaria prevalence (high-high) in LIB, SAL, and URC were located near forest edges. Habitats in marsh and forest edges (especially in early stages of settlement) are able to promote malaria transmission by both increasing entomological risk and increasing human–vector contact as a result of human behavioral patterns associated with economical activities [28]. In an urban area in Senegal, for instance, *An. arabiensis* densities and EIR estimates showed a negative correlation with the distance to the marsh edges [29], while in rural villages in south-eastern Tanzania, high densities of malaria vectors were observed to be clustered in peripheral houses [30]. Numerous studies in the Brazilian Amazon also found a relationship between malaria and proximity to forest, finding high densities of larval and adult *An. darlingi* in forest fringes, as well as increased malaria infections in populations living or working near forest edges [31, 32].

With the main malaria vector *An. darlingi* biting predominantly outdoors, it was not surprising to find a trend of the association of men and individuals aged 10–29 years with increased malaria infection. Similar relationships have been detected in many cross-sectional surveys in the Amazon, explained by the increased outdoor-, social-, and forest-related activities of men, adolescents, and young adults, usually when mosquitoes are more active [19, 20, 33]. However, housing without complete walls as the main risk factor for malaria (observed in other studies in the Amazon) [34, 35] and indoor EIRs as high as outdoor EIRs in some villages (at different times) suggests that intradomiciliary transmission is common in some settings. Indeed, accounting for human behavior in villages [17], we estimated that approximately 90% of human exposure to *An. darlingi* at dusk and dawn would occur indoors where LLINs were not available and/or used. Several studies in Africa reported that main vectors are not predominantly endophagic, but they overwhelmingly preferred feeding at times when most humans are indoors [36–39]. This feeding pattern driven by the mosquito preference for humans in the indoor environment has also been observed in coendemic settings for *P. vivax* and *P. falciparum* in Salomon Islands [40] and Colombia [41], which are dominated by vectors that feed predominantly outdoors.

The successful application of different discipline methodologies to understand malaria transmission dynamics in endemic and remote riverine villages in Mazan together with the comprehensive integration of parasitological, vector, and environmental data prospectively collected during 18 months can be underscored as the main strengths of our study. Nevertheless, difficult access to villages together with budget constraints limited the number of collections of *An. darlingi* mosquitoes during the study period, preventing the statistical assessment of the relationship between parasitological and entomological indicators. Similarly, water level data of the Mazan River were not available to relate to those indicators in LIB and VIB; however, the fact that the highest HBR in these villages occurred 2 months after the peak level of Napo River as in SAL and URC, suggests that water levels of the Mazan River follow the same trends as those of the Napo. Moreover, there are logistical limitations to ground exploration for the identification of all potential breeding sites, and the assessment of proximity to them as a malaria risk factor. Finally, we only performed the risk factor analysis and the spatial analysis with prevalence data of March 2016, because the prevalence of other surveys was likely affected by the deployment of test-and-treat interventions.

Our study confirmed higher prevalence and incidence rates associated with higher density of adult *An. darlingi* in the Mazan basin compared with the Napo basin. High microheterogeneity in malaria transmission was observed within the same villages, as a result of interactions between the microgeographic landscape driving diverse conditions for malaria vector development, the housing structure, and human behavior. Considering the evidence for indoor malaria transmission in the study villages, we recommend the delivery and opportune replacement of LLINs to increase coverage and prevention levels to *An. darlingi* exposure, and education interventions to insure their appropriate use and conservation. Local initiatives to improve housing conditions should be encouraged because they are not only able to reduce malaria, but also they improve health and well-being in general. New vector strategies such as spatial repellents [42] and attractive toxic sugar baits [43], capable of altering mosquito feeding behavior and reducing mosquito densities, both indoor and outdoor, can complement the use of LLINs to further reduce malaria transmission. Similarly, community environment-based management, aimed at removing and/or reducing temporary water collections near houses, and larval source management of permanent and large breeding sites, have been found to be effective vector measures in some endemic settings [44, 45]. The feasibility, operability, and effectiveness of new vector control strategies need to be assessed prior to implementation in the heterogeneous Amazon basin landscape.

## Supplementary Data

Supplementary materials are available at *The Journal of Infectious Diseases* online. Consisting of data provided by the authors to benefit the reader, the posted materials are not copyedited and are the sole responsibility of the authors, so questions or comments should be addressed to the corresponding author.

jiaa496_suppl_Supplementary-Material

## Notes

***Acknowledgments.*** We thank all residents and local authorities from riverine villages in Mazan for their enthusiastic participation in the study, as well as all field workers for their dedication during the study. This publication has been possible thanks to the authorization and permits N.0424-2012-AG-DGFFS-DGEFFS from Dirección de Gestión Forestal y de Fauna Silvestre and Dirección General Forestal y de Fauna Silvestre del Ministerio de Agricultura de la República del Perú.

***Author contributions.*** A. R. A., M. M., N. S., and D. G. conceived and designed the study. D. M. G., M. M., M. S., M. G., J. B., F. A., K. A., G. C. E., and K. P. supervised the fieldwork. M. S., J. C. M., and K. P. supervised the laboratory assays. A. R. A., K. A., and G. C. E. contributed to the data management and the consolidation of the fieldwork and laboratory data. A. R. A., D. M. G., and N. S. conducted the analysis. A. R. A. prepared the figures and tables and wrote the first draft of the paper. M. M., D. M. G., N. S., J. E. C., J. V., A. L. C., and D. G. contributed to the result interpretation and writing of the paper. All authors read and approved the final manuscript.

***Financial support***. This work was supported by the Consejo Nacional de Ciencia, Tecnología e Innovación Tecnológica (grant number 008-2014-FONDECYT); the Académie de Recherche et d’Enseignement Supérieur-Commission de la Coopération au Développement of Belgium (grant number ARES-CCD, PRD-Peru 2014–2019 to N. S., A. L. C., and A. R. A.); World Health Organization Special Programme for Research and Training in Tropical Diseases (grant number 201460655 to D. G.); and National Institutes of Health, National Institute of Allergy and Infectious Diseases (grant numbers U19AI089681 to J. M. V. and R01AI110112 to J. E. C.). A. R. A. is a Postdoctoral Researcher of the Fonds de la Recherche Scientifique (FNRS, Belgium).

***Supplement sponsorship***. The supplement is sponsored by TDR, the Special Programme for Research and Training in Tropical Diseases, based at the World Health Organization.

***Potential conflicts of interest.*** All authors: No reported conflicts of interest. All authors have submitted the ICMJE Form for Disclosure of Potential Conflicts of Interest. Conflicts that the editors consider relevant to the content of the manuscript have been disclosed.
