# Supplementary material for: Integrating Parasitological and Entomological Observations to Understand Malaria Transmission in Riverine Villages in the Peruvian Amazon
Source: J Infect Dis. 2021 Apr 27;223(Suppl 2):S99–S110. doi: 10.1093/infdis/jiaa496 (PMC8079135; doi:10.1093/infdis/jiaa496)
Supplement: jiaa496_suppl_Supplementary-Material [file jiaa496_suppl_supplementary-material.docx]

**SUPPLEMENTARY DATA**

**Supplementary text 1. Laboratory procedures**

**Microscopic diagnosis**: Thick and thin blood smears for microscopic diagnosis were stained on two slides with a 10% Giemsa solution. One slide was immediately read in the field site and the second was read the following day at the reference laboratory in Iquitos for diagnosis confirmation. Negative slides were defined as those having no malaria parasites after examining 100 fields, and parasite density in positive slides was estimated assuming a concentration of 8,000 white blood cells (WBC)/μl (Ministerio de Salud del Perú, 2010). Expert quality control was done blindly on all positive slides and 10% of randomly chosen negative slides.

**Quantitative real-time PCR (qPCR)**: DNA was extracted from ~6 mm^2^-pieces of dried blood spots (DBS) using a commercial DNA extraction kit (QIAamp DNA Blood Minikit, Qiagen, Hilden, Germany). A qPCR method targeting the 18s rRNA gene region was applied following the protocol reported by Mangold et al. (Mangold et al., 2005), to obtain melting curves that accurately differentiate *P. vivax* from *P. falciparum*. Diagnosis by qPCR provided by the local research laboratory in Iquitos was based on the result of one qPCR analysis per sample, while the confirmation of an infection provided by the National Research Laboratory in Lima was based on the results of two qPCR analyses per sample. An infection was confirmed with only one positive qPCR which identified either *P. vivax*, *P. falciparum* or mixed infection. In the case of two positive qPCRs with different species identification, the infection was considered to be a mixed infection.

**Supplementary table 1. Study villages in Mazan**

| **Village** | **River** | **Availability** | | **Time to health centre** | **Houses** | **Population** | **First survey** | **Second survey** | **Third survey** |
| --- | --- | --- | --- | --- | --- | --- | --- | --- | --- |
|  |  | **Health post** | **Community health worker** | **Minutes** | **n** | **N** | **n** | **n** | **n** |
| Libertad | Mazan | Yes | Yes | 180 | 60 | 297 | 230 | 238 | 211 |
| Visto Bueno | Mazan | No | Yes | 360 | 15 | 60 | 51 | 55 | 49 |
| Salvador | Napo | No | Yes | 45 | 77 | 374 | 327 | 319 | 318 |
| Urco Miraño | Napo | No | No | 120 | 53 | 284 | 215 | 231 | 222 |

**Supplementary table 2. Sociodemographic data and malaria preventive measures in study villages**

|  | **Libertad** | **Visto Bueno** | **Salvador** | **Urco Miraño** |
| --- | --- | --- | --- | --- |
|  | (N=297) | (N=60) | (N=374) | (N=284) |
| **Age group (years)*** |  |  |  |  |
| - <10 | 92 (31.0%) | 23 (38.3%) | 147 (39.3%) | 77 (27.1%) |
| - 10-29.9 | 95 (32.0%) | 20 (33.3%) | 96 (25.7%) | 103 (36.3%) |
| - >=30 | 110 (37.0%) | 17 (28.3%) | 131 (35.0%) | 104 (36.6%) |
| **Gender** |  |  |  |  |
| - Female | 140 (47.1%) | 24 (40.0%) | 184 (49.2%) | 131 (46.1%) |
| - Male | 157 (52.9%) | 36 (60.0%) | 190 (50.8%) | 153 (53.9%) |
| **Education*** |  |  |  |  |
| - None | 11 ( 3.8%) | 1 ( 1.7%) | 10 ( 2.8%) | 8 ( 2.9%) |
| - Primary school | 243 (85.0%) | 53 (88.3%) | 321 (88.4%) | 191 (68.5%) |
| - Secondary or higher | 32 (11.2%) | 6 (10.0%) | 32 ( 8.8%) | 80 (28.7%) |
| **Occupation*** |  |  |  |  |
| - Housewife/student/none | 90 (30.5%) | 23 (39.0%) | 102 (27.6%) | 121 (43.4%) |
| Other | 82 (27.8%) | 15 (25.4%) | 123 (33.2%) | 71 (25.4%) |
| - Forest related (farming, logging) | 123 (41.7%) | 21 (35.6%) | 145 (39.2%) | 87 (31.2%) |
| **Malaria in the past year*** |  |  |  |  |
| - No | 194 (66.2%) | 30 (50.0%) | 205 (55.7%) | 105 (37.9%) |
| - Yes | 99 (33.8%) | 30 (50.0%) | 163 (44.3%) | 172 (62.1%) |
| **At least one LLIN for two people*** |  |  |  |  |
| - No | 167 (56.2%) | 19 (31.7%) | 217 (58.0%) | 150 (52.8%) |
| - Yes | 130 (43.8%) | 41 (68.3%) | 157 (42.0%) | 134 (47.2%) |
| **Sleep under LLIN the previous night** |  |  |  |  |
| - No | 72 (24.7%) | 11 (18.6%) | 91 (24.7%) | 54 (19.5%) |
| - Yes | 220 (75.3%) | 48 (81.4%) | 157 (75.3%) | 223 (80.5%) |
| **Indoor residual spraying (past year)*** |  |  |  |  |
| - No | 48 (16.4%) | 21 (35.0%) | 297 (79.4%) | 38 (13.4%) |
| - Yes | 244 (83.6%) | 39 (65.0%) | 77 (20.6%) | 246 (86.6%) |

* *p*<0.05

**Supplementary table 3. Household characteristics reported by study participants**

|  | **Urco Miraño** | **Visto Bueno** | **Libertad** | **Salvador** |
| --- | --- | --- | --- | --- |
|  | (N=284) | (N=60) | (N=297) | (N=374) |
| **Number of external walls*** |  |  |  |  |
| - None | 51 (18.0%) | 9 (15.0%) | 38 (12.8%) | 50 (13.4%) |
| - One-three | 110 (38.7%) | 25 (41.7%) | 181 (60.9%) | 177 (47.3%) |
| - Four | 123 (43.3%) | 26 (43.3%) | 78 (26.3%) | 147 (39.3%) |
| **Wall material*** |  |  |  |  |
| - No walls | 51 (18.0%) | 9 (15.0%) | 38 (12.8%) | 50 (13.4%) |
| - Wood | 216 (76.1%) | 41 (68.3%) | 247 (83.2%) | 285 (76.2%) |
| - Pona (palm) or other | 17 ( 6.0%) | 10 (16.7%) | 12 ( 4.0%) | 39 (10.4%) |
| **Floor material*** |  |  |  |  |
| - Wood | 249 (87.7%) | 44 (73.3%) | 280 (94.3%) | 317 (84.8%) |
| - Pona or other | 35 (12.3%) | 16 (26.7%) | 17 ( 5.7%) | 57 (15.2%) |
| **Ceiling material*** |  |  |  |  |
| - Palm leaves | 82 (28.9%) | 60 (100.0%) | 279 (93.9%) | 235 (62.8%) |
| - Tin (calamina) | 202 (71.1%) | 0 ( 0.0%) | 18 ( 6.1%) | 139 (37.2%) |
| **Water supply** |  |  |  |  |
| - Open well | 16 ( 5.6%) | 0 ( 0.0%) | 0 ( 0.0%) | 0 ( 0.0%) |
| - River, rain | 268 (94.4%) | 60 (100.0%) | 297 (100.0%) | 374 (100.0%) |
| **Sanitation facility*** |  |  |  |  |
| - Inside | 9 ( 3.2%) | 0 ( 0.0%) | 0 ( 0.0%) | 0 ( 0.0%) |
| - Outside (latrine, dug) | 95 (33.5%) | 0 ( 0.0%) | 27 ( 9.1%) | 68 (18.2%) |
| - No facility, field | 180 (63.4%) | 60 (100.0%) | 270 (90.9%) | 306 (81.8%) |
| **Electricity available*** |  |  |  |  |
| - No | 111 (39.1%) | 60 (100.0%) | 268 (90.2%) | 162 (43.3%) |
| - Yes | 173 (60.9%) | 0 ( 0.0%) | 29 ( 9.8%) | 212 (56.7%) |

* *p*<0.05

**Supplementary table 4. Indicators of human exposure to *An. darlingi* estimated from matched analysis of mosquito and human behaviour data in study villages***

|  | **Urco Miraño** | **Visto Bueno** | **Libertad** | **Salvador** |
| --- | --- | --- | --- | --- |
|  |  |  |  |  |
| **Proportion biting indoors** | 0.242 | 0.184 | 0.237 | 0.493 |
|  |  |  |  |  |
| **Behaviour-adjusted exposure - Unprotected individual** |  |  |  |  |
| Proportion of vector bites occurring indoors for an unprotected individual | 0.881 | 0.877 | 0.887 | 0.923 |
| Proportion of vector bites occurring while asleep for an unprotected individual | 0.835 | 0.819 | 0.858 | 0.857 |
|  |  |  |  |  |
| **Behaviour- adjusted exposure – LLIN user** |  |  |  |  |
| Proportion of remaining exposure occurring indoors for a protected user | 0.426 | 0.447 | 0.389 | 0.585 |
| Proportion of human exposure occurring while asleep for a protected user of a LLIN | 0.202 | 0.184 | 0.232 | 0.230 |
| Proportion of human exposure occurring indoors but not asleep | 0.224 | 0.263 | 0.157 | 0.355 |
| Proportion of all vector bites prevented by using a LLIN | 0.793 | 0.778 | 0.815 | 0.814 |
|  |  |  |  |  |
| **Behaviour-adjusted exposure - population mean** |  |  |  |  |
| Proportion of exposure prevented by current levels of LLIN use in the population | 0.581 | 0.605 | 0.581 | 0.600 |

*Indicators were estimated using the methodology and spreadsheet proposed by Monroe et al. [1] and published data of hourly mosquito and human behaviour between 18:00-06:00 h in study villages [2]. Human behaviour data included LLIN use the night before, and the time to go sleep and wake up indoors. We assumed similar values for both the proportion of people outside houses and the proportion of people inside them but not sleeping. This assumption could overestimate the number of people outdoors in some hours, especially if electricity is not available. We also assumed a conservative personal protection of 90% when using a LLIN based on a report of the effect of permethrin impregnated bednets on blood-feeding behaviour of *An. darlingi* mosquitoes in the Peruvian Amazon [3].

**References**

1. Monroe A, Moore S, Okumu F, et al. Methods and indicators for measuring patterns of human exposure to malaria vectors. Malar J. **2020**; 19(1):207.
2. Saavedra MP, Conn JE, Alava F, et al. Higher risk of malaria transmission outdoors than indoors by *Nyssorhynchus darlingi* in riverine communities in the Peruvian Amazon. Parasit Vectors. **2019**; 12(1):374.
3. Zamora-Perea E, Orellana Rios W, Curto E, et al. Entomological evaluation of permethrin impregnated bednets against *Anopheles darlingi* in the Peruvian Amazon. Am J Trop Med Hyg. **2008**; 79 (Suppl.): 226.
